# Platelet-Rich Plasma and Adipose-Derived Mesenchymal Stem Cells in Association with Arthroscopic Microfracture of Knee Articular Cartilage Defects: A Pilot Randomized Controlled Trial

**DOI:** 10.1155/2022/6048477

**Published:** 2022-04-28

**Authors:** Michele Venosa, Francesco Calafiore, Manuel Mazzoleni, Emilio Romanini, Simone Cerciello, Vittorio Calvisi

**Affiliations:** ^1^Department of Life, Health and Environmental Sciences, University of L'Aquila, Via Vetoio Coppito 2, 67100 - L'Aquila, Italy; ^2^RomaPro Center for Hip and Knee Arthroplasty - Polo Sanitario San Feliciano, Via Mattia Battistini, 44, 00167 - Rome, Italy; ^3^Polo Sanitario San UOSD, Department of Mini-invasive and Computer-assisting Orthopaedic Surgery, San Salvatore Hospital, Via L. Natali 1, 67100 - L'Aquila, Italy; ^4^GLOBE, Italian Working Group on Evidence Based Orthopaedics, Via Nicola Martelli, 3, 00197, Rome, Italy; ^5^Orthopaedic Department, Casa di Cura Villa Betania, Via Pio IV, 42, 00165 - Rome, Italy; ^6^Orthopaedic Department, Marrelli Hospital, Via Gioacchino da Fiore, 5, 88900 - Crotone, Italy

## Abstract

**Background:**

This study aims to compare the effects of platelet-rich plasma (PRP) alone or in combination with adipose-derived mesenchymal stem cells (AD-MSCs) in patients affected by cartilage defects, undergoing knee arthroscopic microfracture.

**Methods:**

Thirty-eight patients diagnosed with a knee monocompartmental cartilage defect (Outerbridge grade IV) on the MRI, underwent an arthroscopic procedure. After the confirmation of the lesion, they all received the same bone marrow stimulation technique (microfracture) and were randomized into two groups: the first one had additional PRP injection (group A), while the second received PRP and AD-MSC injection (group B). Knee assessment and pain score were documented with Knee Injury Osteoarthritis Outcome Score (KOOS), International Knee Documentation Committee (IKDC) score, Short-Form (SF) 12, and Visual Analogue Scale (VAS) before the treatment and at 1, 3, 6, and 12 months of follow-up postoperatively. An additional arthroscopic procedure, performed in four patients for a subsequent meniscal lesion, let us evaluate cartilage evolution by performing a macro/microscopical assessment on cartilage biopsy specimens.

**Results:**

At the 12-month follow-up, both groups showed a comparable functional improvement. The scores on the IKDC form, KOOS, pain VAS, and SF-12 significantly improved from baseline (*p* < 0.05) to 12 months postoperatively in both treatment groups. The four second-look arthroscopies showed a complete repair of the articular defects by smooth solid cartilage layer, with a good chondrocytic population, in both groups. A thick smooth hyaline-like cartilage with a predominantly viable cell population and normal mineralization (a form closely resembling native tissue) was observed in group B.

**Conclusions:**

Modern regenerative medicine techniques, such as PRP and AD-MSC, associated with traditional arthroscopic bone marrow stimulating techniques, seem to enhance cartilage restoration ability. The preliminary results of this pilot study encourage the synergic use of these regenerative modulating systems to improve the quality of the regenerated cartilage.

## 1. Introduction

Articular cartilage is a specific connective tissue formed of hyaline avascular, aneural, and alymphatic cartilage. Its metabolism depends on nutrients diffusion from the synovial fluid; for this reason, injury responses originating from the chondrocytes themselves are initially predominant [1].

Chondral lesions may develop as a consequence of mechanical deformity and/or joint instability, and they are frequently observed with a high prevalence in both the general population and high-demand athletes. Focal chondral defects occur in up to two-thirds of patients undergoing knee arthroscopy [2], and they are often asymptomatic so that careful assessment is required [3]. The etiology and pathogenesis of many cartilage diseases are not fully understood. They are commonly encountered in orthopedic practice, since articular cartilage has a poor self-regeneration ability, due to the absence of vascularization and proliferation of chondrocytes.

Several conservative options have been proposed to reduce pain and restore articular function; besides a wide array of surgical techniques (abrasive chondroplasty, microfracture, and spongialisation) have been proposed for the management of chondral lesions [3, 4].

Arthroscopic microfracture is a single-stage cheap arthroscopic procedure requiring no special instrumentation, intended to stimulate cartilage regeneration, although the histological quality and endurance of the repaired cartilage considerably limit the results, which are only temporary and better for low-grade chondral lesions.

An appropriate environment with specific growth factors is a basic condition for the development of hyaline-type repair cartilage.

Platelet-rich plasma (PRP) has been widely used in the last decades as an ideal option for cartilage defects since it is an important source of growth factors and cytokines able to promote cartilage healing, improve clinical function and reduce pain [5]. Based on a consistent background of science studies, PRP has gained great popularity in the last years and it is becoming a promising modern regenerative approach in the treatment of articular chondral lesions even though clinical results tend to lessen over time [6–11].

Mesenchymal stems cells (MSCs) or medicinal signaling cells as Caplan referred [12] have recently emerged as a promising regenerative option. They are now widely used experimentally for cartilage-tissue engineering with promising results, having been previously investigated and appreciated for their accessibility, their immunomodulation action, their ability for self-renewal, and their chondrogenic capability.

MSCs found in the synovial membrane after intra-articular injection act to establish a regenerative microenvironment at the site of the articular defect and express molecules with anti-inflammatory and chondrogenic capabilities [13]. These cells could enhance the activation and differentiation of endogenous stem cells with the potential to repair the articular cartilage [14].

The association between PRP and MSCs is an experimental alternative approach for the treatment of chondral lesions in consideration of their synergic action, based on the ability of platelets growth factors to improve the reparative properties of the CD34^+^ precursor cells seeded at the defect.

This randomized prospective clinical trial aimed to report the effects of PRP alone or in combination with adipose-derived MSCs (AD-MSCs) in patients undergoing arthroscopic microfracture for knee monocompartmental cartilage defects (Outerbridge Grade IV): the rationale behind this concept was to stimulate a spontaneous repair reaction, although experience has shown that the new cartilage is variable in structural composition, quality, and durability [15].

## 2. Materials and Methods

A consecutive series of 38 patients (mean age 56.3 years; range: 45–73 years) were enrolled in this monocentric prospective randomized controlled trial (with two treatment arms) performed between May 2019 and December 2019. It was not possible to blind the patients as to what treatment they have received, since one treatment arm consists of a two-stage surgery, including a miniabdominal liposuction to remove adipose tissue and isolate AD-MSCs.

All patients had a diagnosis of knee monocompartmental cartilage defect (Outerbridge grade IV) on the MRI confirmed during a subsequent arthroscopic procedure. The patients had to be 18–75 years of age and symptomatic. Exclusion criteria were either local or general. Local factors included evidence of radiographic knee or hip osteoarthritis, flexion deficit >20°, extension deficit >5°, knee alignment >5° in varus and valgus, major ligament injury or knee instability, cartilage defects on the patellofemoral joint or in contralateral knee compartment, previous surgery to the chondral defect, and meniscal lesions. All cartilage lesions were inferior to 2 cm^2^ at arthroscopic evaluation that was performed within 3 months from the onset of the symptoms in all cases.

**Figure 1:**
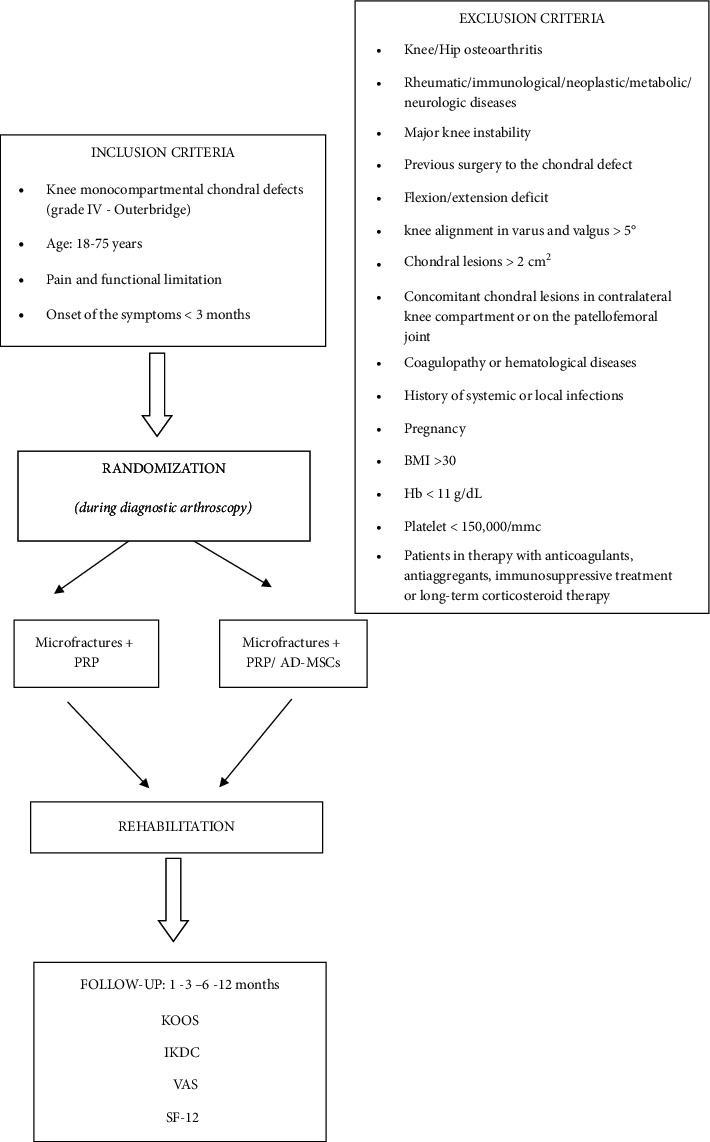
Flow chart: overview of the study.

General factors included rheumatic/immunological/neoplastic/metabolic/neurologic/hematological diseases, history of systemic or local infections, patients in therapy with anticoagulants or antiaggregants, patients with Hb values < 11 g/dL and platelet values < 150,000/mmc, severe cardiovascular diseases, pregnancy, body mass index (BMI) > 30, and patients receiving immunosuppressive treatment or long-term corticosteroid therapy (Figure 1).

The study protocol was approved by the Internal Review Board of the authors' affiliated institutions and was consistent with the ethical principles for medical research ratified by the World Medical Association (WMA) Declaration of Helsinki. All patients participating in the study were carefully informed about the modality and purpose of this research, and they subscribed to a specific informed consent form.

All patients, preliminary evaluated with knee MRI [16] and clinical examination by the senior author, underwent diagnostic arthroscopic evaluation and concomitant bone marrow stimulation technique; cartilage underwent arthroscopic debridement with an arthroscopic abrader to form a stable edge of healthy cartilage [17] and microfracture with angled pics to stimulate cartilage response. At the end of the procedure, all patients were randomized into two groups: 19 patients (group A) were additionally treated with PRP injection whereas the other 19 patients (group B) were additionally treated with PRP injection and autologous AD-MSCs infiltration. Groups characteristics at inclusion are shown in Table 1. Patients were assigned to one of the two groups (allocation ratio 1 : 1) using sequentially numbered sealed envelopes safeguarded by an orthopedic resident (“randomization assistant”), who was the only member of the staff with access to the allocation spreadsheet during the trial.

Four patients (2 for group A and 2 for group B) required a further arthroscopic treatment one year later for a subsequent traumatic meniscal lesion, giving us the opportunity of evaluating cartilage evolution by performing a macro/microscopical assessment on cartilage biopsy specimens. Histological evaluation refers to the International Cartilage Repair Society (ICRS) visual histological assessment scale (Table 2).

Baseline demographic data was collected before treatment. Patients were prospectively evaluated before surgery and at 1, 3, 6, and 12 months of follow-up using KOOS (Knee Injury Osteoarthritis Outcome Score), IKDC (International Knee Documentation Committee), SF-12 (Short-Form 12) for general health status, and VAS (visual analogue scale) for pain evaluation.

All clinical evaluations were performed by a medical member of the staff not involved in the surgical/injective procedure so that follow-up was blinded and the examiner was unaware of which treatment arm the patient belonged to (the patients were instructed not to reveal the nature of the treatment received).

Adverse events (pain and swelling with longer recovery time) were observed in 5 patients with a kissing lesion (2 for group A and 3 for group B) and treated with rest, ice and oral nonsteroidal anti-inflammatory drugs (NSAIDs).

### 2.1. Statistical Analysis

Demographic and clinical characteristics have been presented as means and standard deviations (SD) for data with normal distribution and median and interquartile range for nonparametric data.

*p* values of <0.05 were considered to indicate the statistical significance, and they were interpreted based on Bonferroni correction. All baseline data were compared between groups by chi-square, *t*-test, and Mann–Whitney *U* tests. All statistical analyses were performed using MedCalc® version 13.3.1.

### 2.2. Sample Size

Sample size calculation is not possible considering the insufficient data currently available on the effects of PRP and AD-MSCs on knee chondral defects. Pilot studies, as is well known, should be undertaken to allow trial protocols to be tested under study conditions before evaluation in a full randomized controlled trial, according to published guidelines. Based on this precondition, we recruited 38 participants in this pilot study with 19 patients allocated to each group.

## 3. Results

**Figure 2:**
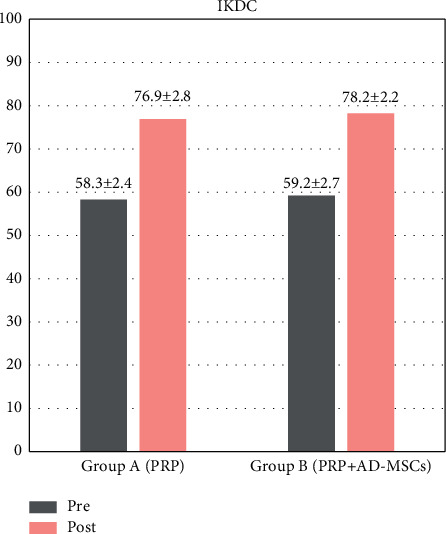
IKDC (International Knee Documentation Committee) score: pretreatment and at 12-months follow-up.

SF-12, VAS, IKDC, and KOOS scores were collected for all patients preoperatively and at 1, 3, 6, and 12 months of follow-up. Mean IKDC scores for group A and group B at baseline and at the final follow-up are shown in Figure 2. No significant differences in mean IKDC scores were detected between PRP/AD-MSC-treated patients and PRP alone-treated patients. The mean IKDC score at 12-months follow-up in group A was 76.9 ± 2.8 compared to 78.2 ± 2.2 in group B. The increase in IKDC score from baseline to follow-up was significant for both groups.

**Figure 3:**
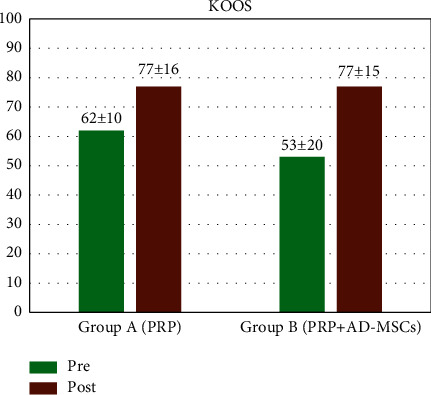
KOOS (Knee Injury Osteoarthritis Outcome Score): pretreatment and at 12-months follow-up.

The KOOS profiles with mean scores before surgery and at 12 months of follow-up for group A and group B are shown in Figure 3. There were no differences between the two groups in any of the KOOS subscales at follow-up (group A: 77 ± 16; group B: 77 ± 15) with a slight improvement from baseline in group B compared to group A (preoperative scores: group A: 62 ± 10; group B: 53 ± 20).

**Figure 4:**
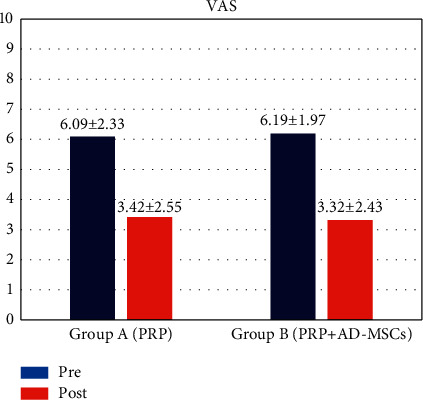
VAS (visual analogue scale) score: pretreatment and at 12-months follow-up.

**Figure 5:**
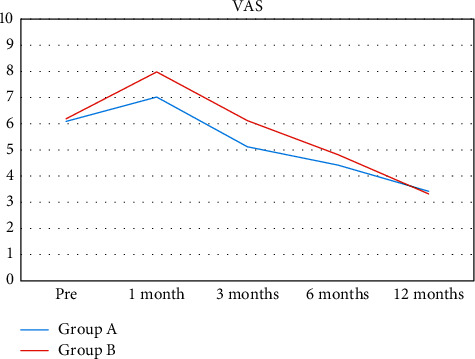
VAS (visual analogue scale) score evolution over time in the two cohorts: repeated VAS score analyses showed a significant and comparable reduction of pain intensity for both groups started one month after surgery.

**Figure 6:**
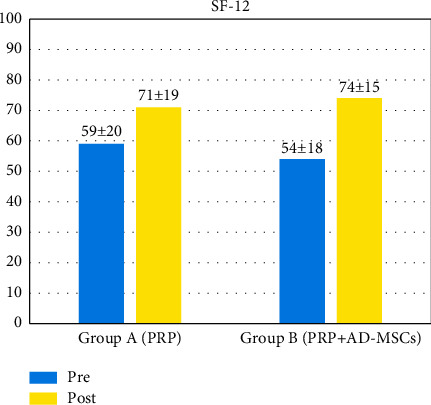
SF-12 (Short-Form 12) score: pretreatment and at 12-months follow-up.

In both groups, we appreciated a similar enhancement from baseline in VAS (Figure 4 and Figure 5) and SF-12 scores (Figure 6). VAS score dropped from 6.09 ± 2.33 before treatment to 3.42 ± 2.55 at 12 months of follow-up for group A and from 6.19 ± 1.97 to 3.32 ± 2.43 for group B. SF-12 scores reported a similar progression for both groups with a significant functional enhancement after regenerative medicine and surgery treatment and better results after PRP/AD-MSC association treatment (group A: from 59 ± 20 to 71 ± 19; group B: from 54 ± 18 to 74 ± 15).

**Figure 7:**
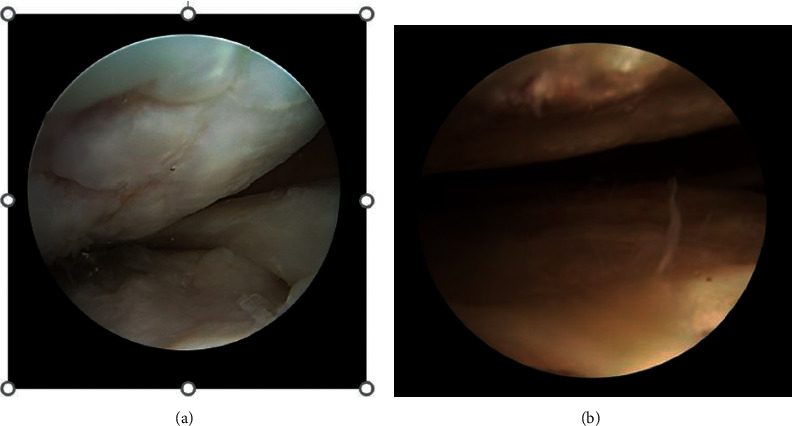
Intraoperative second-look arthroscopic images of cartilage repair sites (after 12 months). (a)The original chondral lesion is filled with fibrocartilagineous tissue after microfractures + PRP (platelet-rich plasma) injection. (b) A hyaline-like cartilage filling the chondral defect is detectable after microfractures + PRP + AD-MSC (adipose-derived mesenchymal stem cells) treatment.

The additional arthroscopic procedures performed one year after regenerative/arthroscopic treatment in 4 patients (2 patients for group A and 2 patients for group B) affected by a subsequent traumatic meniscal lesion showed a complete restoration of the chondral defects, with smooth hyaline cartilage in all patients, and findings consistent with clinical outcomes. With a probe, we appreciated a firm chondral surface like healthy articular cartilage (Figure 7). Macroscopic and microscopic histological evaluations of biopsy samples were performed according to ICRS visual histological assessment scale; all data are reported in Table 3. The arthroscopic examination revealed a smooth resurfacing fibrocartilage with predominantly viable cell population and abnormal cartilage mineralization in group A; a thick smooth hyaline-like cartilage with predominantly viable cell population and normal mineralization in group B.

## 4. Discussion

The results of the present study showed progressive pain reduction and satisfying functional recovery in both groups. However, no significant statistical difference was observed between group A (microfracture + PRP) and group B (microfracture + PRP + AD-MSCs) in terms of clinical outcomes (KOOS, IKDC, SF-12 and pain VAS) at 1-year follow-up. The most relevant aspect of the study is related to the histological evidence: the association of PRP and AD-MSCs seemed to stimulate the qualitative improvement of the regenerated cartilage, with histological properties closely resembling native articular cartilage. Anyway, these findings should be carefully interpreted according to the evident statistical limits of the study.

Repair and regeneration of articular cartilage is a significant challenge for orthopedic surgeons: traditional stimulation treatments (microfractures or osteochondral substitutes) can enhance knee function by stimulating a spontaneous repair reaction but they cannot restore a cartilage structure histologically resembling native tissue [4, 18, 19]. The tissue formed is variable in composition and durability. These treatment modalities are usually unsatisfactory in the long term, and eventually fail since fibrocartilage develops in the long term, rather than the desired hyaline cartilage [4, 20].

Microfracture is a consolidated arthroscopic technique, widely performed in the last 30 years in the treatment of full-thickness cartilage defects to assist in stimulating a healing response. It is cost-effective and not technically demanding, with an extremely low rate of patient morbidity. Moreover, it does not compromise the possibility of further additional surgeries [21, 22] though a recent systematic review by Cogan et al. demonstrated a higher failure rate for patients treated with autologous chondrocytes implantation (ACI) after a prior bone marrow stimulation technique (like microfracture) [23]. Moreover many studies demonstrated that the quality of cartilage repair tissue and the clinical outcomes are significantly better for patients with smaller chondral lesions [24–26]; therefore, the bigger chondral lesions should be addressed by recurring to more aggressive therapies. Another important consideration is the time elapsed from the injury since chondral lesions treated within 12 weeks from injury show better clinical results [15]. Age affects the outcome of microfracture: clinical evidence shows that younger patients (<40 years) might benefit more from this bone marrow stimulating technique [26–28]. Absolute contraindications for microfracture include: infections, inability to perform/attend to rehabilitative protocols, end-stage osteoarthritis or lesions bigger than 2 cm^2^; relative contraindications are knee malalignment, ligamentous instability, BMI >35 kg/m^2^, meniscal insufficiency, corticosteroid therapy, and smoking [29]. A lower-limb malalignment can accelerate the articular cartilage wear and can invalidate postsurgical outcomes; for this reason, adequate overall surgical planning (taking into account a potential malalignment correction) should be addressed before performing a regenerative treatment focusing on chondral lesions.

The rationale behind microfracture is to expose bone marrow-derived pluripotent cells to the articular surface so that, as reported by Steadman, “*microfracture does not lead to tissue replacement; rather, the microfracture procedure relies on a “marrow-based strategy” for tissue repair”* [4].

Based on this evidence, we hypothesized to improve this “marrow-based strategy” by taking advantage of PRP and AD-MSCs properties.

Chondral lesions are usually characterized by mechanical and oxidative stress with the recruitment of inflammatory cells and proinflammatory cytokine production (IL-1, IL-6, and TNF-*α*). For this reason, several studies have focused on the anti-inflammatory and immunomodulatory properties of PRP in the treatment of articular cartilage defects. The *α*-granules of the concentrated platelet-rich plasma contain several growth factors as platelet-derived growth factor (PDGF), vascular endothelium growth factor (VEGF), insulin-like growth factor (IGF), fibroblast growth factor (FGF), epidermal growth factor (EGF), and tissue growth factor (TGF) [11], able to activate tissue regeneration and modulate the whole joint environment thanks to their anti-inflammatory properties [6, 8]. Furthermore, PRP indirectly induces the activity of transcription factor NF-*κ*B and reduces nitric oxide (NO) levels [30]. NO increases the production of metalloproteinases, and it is responsible for chondrocyte apoptosis, thus contributing to cartilage degeneration. On this basis, PRP has demonstrated promising results for the treatment of chondral defects, and the evidence of clinical efficacy has been appreciated through multiple studies [8, 9, 31].

On this premise, PRP can be considered an optimal solution in the treatment of cartilage lesions if it is used as an adjunct during surgery. In 2013, Lee et al. compared the outcomes of microfracture and microfracture associated with PRP injection in patients affected by focal chondral lesions [32]. Significant superior outcomes were reported in patients who had undergone microfractures and PRP at 2-year follow-up. These results were confirmed by a more recent RCT performed by Papalia et al. who reported improved clinical outcomes when microfracture was associated with PRP injection (performed either intraoperatively or postoperatively) [33]. In a recent study published by Danieli et al., the authors reported better clinical outcomes for chondroplasty and PRP injection compared with chondroplasty alone in patients affected by ICRS grade III knee chondral lesions at 2-year follow-up [34]. Thanks to its anti-inflammatory properties PRP has been proposed also in the treatment of patients affected by knee osteoarthritis, with better results in patients with low-grade involvement (Kellgren–Lawrence grade I-II). Only minimal clinical improvements have been observed in severe osteoarthritis so that PRP is not recommended in these patients [31, 32]. Anyway, no conclusive evidence has been reported at the moment.

MSCs act in a paracrine way as pericytes can enhance angiogenesis and inhibit apoptosis [35]. They have not only anti-inflammatory properties but also a regenerative role, thanks to a direct cell-to-cell interaction [36]. In the last years, many experimental basic science strategies focused on MSC's function as candidates for use in the regeneration of articular cartilage. MSCs are readily available in large quantities since they can be isolated from a wide array of tissues with no significant collateral morbidity: adipose tissue is an optimal source. The immunomodulation function of MSCs is mediated through the secretion of the so-called exosomes, extracellular vesicles with a diameter range of 30–150 nm, and the release of multiple growth factors with synergic activity [37]. Although the mechanisms underlying cartilage regeneration promoted by MSC exosomes have not been completely clarified, they are widely considered as the principal therapeutic actors in tissue regeneration [38]. MSCs can be used as an adjuvant during surgical procedures. Koh et al. showed better MRI cartilage appearance after microfracture and AD-MSCs injection compared to microfracture alone in patients with focal chondral defect at 2-year follow-up [39]. Hashimoto et al. in their RCT compared the outcomes of arthroscopic bone marrow-derived MSCs injection with microfracture versus microfracture alone, in patients affected by knee chondral defects. The association of MSCs/microfracture was more effective, providing a better quality of articular cartilage surface and better KOOS QOL scores [40].

Recently, Qiao et al. compared the outcomes of microfractures in combination with saline solution injection or hyaluronic acid (HA) injection or adipose-derived mesenchymal progenitor cells injection plus HA in patients with knee cartilage defects. They reported better clinical function (without adverse events) at 2-years follow-up in the third group, as demonstrated by WOMAC and SF-36 measures. The clinical results were consistent with the radiological, arthroscopic and histological findings which confirmed that this treatment can promote chondral defect reduction and cartilage regeneration [41].

Previous studies in the last decades have indicated the potential benefit of PRP and AD-MSC therapy in symptomatic knee cartilage defects. The results of this pilot study indicate that both modalities are safe and effective with the ability to modify disease progression and enhance overall function. In both groups, a significant pain reduction in association with enhanced functional abilities has been observed. In our opinion, a key factor of the study is the histological evaluation of biopsy samples (although the evident statistical limits) that has shown encouraging results even in terms of microscopic appearance, especially in patients treated with the association PRP/AD-MSC.

The present study has some notable limitations. First of all, the small number of patients in both groups. A larger-scale study would be necessary for a more powerful clinical application. Second, the short-term follow-up may be ineffective to investigate the late evolution of the clinical outcomes. Further studies with longer follow-ups would be beneficial. Third, it would be useful to investigate whether multiple PRP or PRP/MSCs injections may have additional effects over a single injection in terms of pain reduction and function improvement. Fourth, an optimal rehabilitation schedule needs to be further investigated. Although aware of these limitations the present study may be a pilot for further additional research and clinical projects.

## 5. Conclusions

Chondral defects treatment is still a challenge for orthopedic surgeons, but over the last decades, regenerative medicine has opened the curtain wide for promising results. PRP and PRP and MSCs have been proposed with encouraging results. According to the present study, no statistically significant difference came to light in terms of clinical outcomes (IKDC, KOOS, SF12, and VAS) between the two cohorts. However, the regenerated cartilage showed more hyaline-like features (higher collagen content, increased mineralization degree, and a higher number of viable cells) in the PRP + MSCs group. This trend should be confirmed in further studies since it may result in superior clinical outcomes at longer follow-up.

## Figures and Tables

**Table 1:** Demographic characteristics of the two cohorts: group A: microfractures + PRP injection; group B: microfractures + PRP and AD-MSC injections.

Characteristics	Group A (microfractures + PRP)	Group B (microfractures + PRP + AD-MSC)
Mean age	56.4 y	55.8 y
Sex (i) Male (ii) Female	12 7	9 10
BMI	26.2 ± 2.6	25.8 ± 3.1
Affected side (i) Right (ii) Left	13 6	14 5

**Table 2:** ICRS (International Cartilage Research Society) Visual Histological Assessment Scale.

Feature	Points
Surface Smooth/continuous Discontinuities/irregularities	3 0
Matrix Hyalin mixture: hyaline/fibrocartilage Fibrocartilage Fibrous tissue	3 2 1 0
Cell distribution Columnar Mixed/columnar-clusters Clusters Individual cells/disorganized	3 2 1 0
Cell population viability Predominantly viable Partially viable <10% viable	3 1 0
Subchondral bone Normal Increased remodelling Bone necrosis/granulation tissue Detached/fracture/callus at base	3 2 1 0
Cartilage mineralization (calcified cartilage) Normal Abnormal/inappropriate location	3 0

**Table 3:** Histologic features of the bioptic samples according to ICRS Visual Histological Assessment Scale (group A: PRP; group B: PRP + AD-MSC).

	Group A		Group B	
Surface	3	3	3	3
Matrix	2	1	3	3
Cell distribution	1	2	2	3
Cell population viability	1	1	3	3
Subchondral bone	2	2	3	2
Cartilage mineralization	0	0	3	3

## Data Availability

The data used to support the findings of this study are available from the corresponding author upon request.
